# Comparative Analysis of Abattoir-Based Measures and On-Farm Pig Welfare Indicators in Italian Fattening Heavy Pigs

**DOI:** 10.3390/vetsci13040361

**Published:** 2026-04-08

**Authors:** Lucia Scuri, Matteo Recchia, Federico Scali, Claudia Romeo, Antonio Marco Maisano, Giovanni Santucci, Camilla Allegri, Marta Masserdotti, Miriam Tenuzzo, Adriana Ianieri, Sergio Ghidini, Giovanni Loris Alborali

**Affiliations:** 1 Department of Food and Drug, Parma University, 43126 Parma, Italy; lucia.scuri@ats-brescia.it (L.S.); adriana.ianieri@unipr.it (A.I.); 2 Istituto Zooprofilattico Sperimentale della Lombardia e dell’Emilia-Romagna ‘Bruno Ubertini’ (IZSLER), 25124 Brescia, Italy; matteo.recchia@izsler.it (M.R.); federico.scali@izsler.it (F.S.); claudiarosa.romeo@izsler.it (C.R.); antoniomarco.maisano@izsler.it (A.M.M.); giovanni.santucci@izsler.it (G.S.); marta.masserdotti@izsler.it (M.M.); miriam.tenuzzo@izsler.it (M.T.); giovanni.alborali@izsler.it (G.L.A.); 3 Department of Veterinary Medicine and Animal Sciences, University of Milan, 26900 Lodi, Italy; sergio.ghidini@unimi.it

**Keywords:** iceberg indicators, slaughterhouse, carcass lesions, tail injuries, wounds on the body, ear injuries, animal welfare, swine, intensive farming

## Abstract

Animal welfare monitoring is essential in pig production. On-farm animal welfare (AW) assessments may provide a comprehensive overview but are resource-intensive. Abattoir-based assessments allow pigs from multiple farms to be inspected in a single facility. However, data on the relationship between these assessments remain limited, especially for heavy pigs (160–170 kg). This study investigates these associations in Italian heavy pig production. At the abattoir, 18,333 pig carcasses from 185 batches across 86 farms were scored for tail, skin (cranial and caudal) and ear lesions. On-farm AW assessments (management, structures and animal-based measures) were obtained from the national surveillance system (ClassyFarm). Tail lesion scores were higher in pigs with intact tails, whereas ear scores showed the opposite trend, suggesting a substitution effect between tail and ear biting. This indicates that tail docking is insufficient to fully prevent abnormal behaviours. Higher skin and ear scores were associated with suboptimal management, but tail scores were not, likely due to the multifactorial nature of tail biting. Herd size had no significant effect on welfare indicators. These results highlight the complexity of assessing AW and the importance of combining abattoir and farm data to obtain a more integrated monitoring system.

## Simple Summary

On-farm evaluations provide direct insight into animal welfare, but they are costly and time-consuming. Assessing key indicators (e.g., tail, skin and ear lesions) at the abattoir improves efficiency by centralising the examination of pigs from multiple farms. However, agreement between these methods remains underexplored, particularly in heavy pig production (160–170 kg). This study examined over 18,000 heavy pig carcasses from 86 Italian farms, comparing abattoir-derived welfare indicators with on-farm assessments (management, structures and animal-based measures). Pigs with undocked tails had more tail lesions, while docked pigs had more ear lesions, which were also associated with traumatic skin lesions. Skin and ear injuries were linked to suboptimal management. Conversely, implementing optimal management practices effectively reduced the occurrence of these lesions. These results confirm that tail docking does not fully prevent aggressive behaviour, as pigs may bite other body parts. The relationship between abattoir and on-farm assessments for tail lesions was less evident, likely due to their complex causes. Integrating abattoir findings with on-farm assessments can support farmers and veterinarians in improving animal welfare monitoring in a more coordinated and feasible way.

## 1. Introduction

In recent decades, animal welfare (AW) has become a key issue in pig production, driven by both growing ethical concerns and stricter regulatory requirements (Directive 98/58/EC). The promotion of AW is crucial to ensure public health and environmental sustainability. Indeed, improving AW has both direct and indirect impacts on public health by reducing the risk of zoonotic disease transmission [1], contributing to the control of antimicrobial resistance [2] and improving food safety [1]. Regulation (EU) 2017/625 defines the legislative framework for official controls within the European Union, including AW, and supports the adoption of standardised evaluation methods.

Over the years, several methods have been proposed to assess AW in pig farms [3,4,5]. In 2018, the Italian Ministry of Health launched ClassyFarm, a national surveillance system for animal production that covers several topics, including AW. This system integrates data from several sources, including self-monitoring and official controls (https://www.classyfarm.it, accessed on 13 March 2026). The ClassyFarm system provides standardised questionnaires for the on-farm AW assessment, covering resource-based and animal-based measures (ABMs). Resource-based measures evaluate management, personnel, environment, facilities and emergency plans, in the light of legal requirements (Legislative Decree n. 146/2001; Legislative Decree n. 122/2011), while ABMs are indicators related to the animals’ experience and ability to cope with the environment [4]. Although on-farm AW assessment protocols are considered to be reliable and repeatable, their application in pig farms can be costly and time-consuming. Moreover, the ABMs assessment could be biassed by high stocking densities, dirtiness and dim lighting [6].

In the European Union, all animals intended for slaughter must undergo an inspection under the responsibility of Official Veterinarians to ensure that the meat is fit for human consumption (Regulation (EU) 2017/625; Regulation EU 2019/627). Integrating AW evaluations into pre-existing inspection activities at the abattoir offers several advantages as it allows the assessment of a larger number of animals from different farms, thereby reducing the costs and resources required for on-farm monitoring while simultaneously mitigating biosecurity risks [7,8]. Therefore, while abattoirs remain of primary importance for public health protection, they are now also regarded as strategic observatories for animal health and welfare monitoring [9,10,11]. The information collected at the abattoir can integrate and support the evaluations carried out on the farm, providing constructive feedback to farmers and herd veterinarians and allowing for farm benchmarking [12,13]. For these reasons, the Italian Ministry of Health has already planned to include data collected at the abattoir in the ClassyFarm surveillance system.

The Farm Animal Welfare Council (FAWC) has recommended the use of ‘iceberg indicators’ of AW at the pig abattoir, suggesting a higher prevalence of lesions in a batch might indicate multiple underlying welfare issues at the farm of origin [14]. These indicators can be easily measured during ante-mortem or post-mortem inspections [15] and provide information on the overall welfare status of a given population [5].

Tail and traumatic skin lesions are well-known ‘abattoir-based measures’ for assessing the welfare of finishing pigs [11,16]. Such lesions, whether acquired early or later in life, tend to remain visible on the carcass at the time of slaughter and can effectively reflect the lifetime welfare status of the animals [6]. Tail lesions range from bruising to mild bite marks, with or without loss of skin integrity, up to a complete loss of the tail [5]. They typically result from tail biting, a multifactorial abnormal behaviour severely impacting AW and herd profitability [16,17]. The practice of tail docking can significantly reduce tail biting and cannibalism [18]. However, these phenomena are often the result of management and structural deficiencies such as pen density, lack of enrichment material or other environmental stressors, which can be masked by such practice [16,19,20]. In Italy, where tail-docking is still widespread, a national plan was launched in 2018 to phase out the practice in order to fully comply with EU legislation (Directive 2008/120/CE).

Traumatic skin lesions are frequently associated with aggressive interactions between pen mates, poorly designed facilities or suboptimal management and handling practices [21]. The main risk factors contributing to traumatic skin lesions include regrouping during the rearing phase, feeding regimes and pen density [22,23,24,25]. However, the development of traumatic skin lesions is a multifactorial problem, with transport and lairage at the abattoir also playing a crucial role [26,27]. Ear lesions are another iceberg indicator of AW [28]. Although numerous risk factors have been proposed for the development of these lesions, no single definitive aetiology has been identified [29,30]. Similar to tail lesions, ear scratches and bruises are often associated with aggression or biting behaviour [28].

The integrated assessment of AW indicators evaluated on-farm and at the abattoir would provide a comprehensive overview of the conditions under which animals are reared. Relationships between these indicators have been explored in a few studies [12,31,32,33,34], while others focused primarily on the effects of transport and of the immediate pre-slaughter period on AW [31,34,35,36,37]. In addition, most of these studies have been carried out on pigs slaughtered at around 100 kg, while data on heavy pigs (160–170 kg) are limited. This study aims to explore the relationships and potential complementarity between abattoir-based iceberg indicators (tail, skin, ear lesions) and farm-level welfare assessments in heavy pig production, both carried out using a well-established surveillance system. This could help to improve the framework for monitoring heavy pig welfare at different stages of production.

## 2. Materials and Methods

### 2.1. Abattoir Evaluations

Abattoir evaluations were carried out in northern Italy between January and October 2023 on randomly selected batches of commercial hybrid heavy pigs intended for the production of Protected Designation of Origin (PDO) ham (160–170 kg and approximately 9–11 months old) at a high-throughput abattoir (480 carcasses/hour). A batch was defined as a group of animals from the same farm slaughtered on the same day. A total of 222 batches from 108 intensive indoor farms were assessed during the study period.

Three abattoir-based measures (tail, skin, and ear lesions) were selected for this study due to their importance and potential as iceberg indicators of AW in fattening pigs [11,16]. Pig carcasses were examined at a distance of approximately one metre after scalding and dehairing, which has been identified as the optimal point for the assessment of tail and traumatic skin lesions in this species [38]. A convenient location was chosen where the carcasses were already split in half. Only the left half of each carcass was examined, being the first visible on the slaughter line. Tail, skin and ear lesions were scored simultaneously by visual inspection by two of the authors (L.S. and M.R.), with one inspecting the carcass while the other recorded the data on a paper sheet. All data were subsequently digitised by the two assessors. The time available to score each animal was determined by the speed of the line (approximately 6–7 s per carcass). The scoring systems for tail, skin and ear lesions are summarised in Table 1 and examples of each score are provided in the Supplementary Materials.

Tail injuries were scored on a scale of 0 to 3 and classified as either intact or non-intact, regardless of the presence of acute lesions [39,40]. A tail was considered intact if it was undocked and the normal anatomy of the tip (i.e., rounded and slightly flattened end) was preserved. A non-intact tail could be either docked or shortened due to healed tissue loss, possibly resulting from a history of tail biting on the farm. In such cases, the tail is scarred, shortened, abnormally shaped or too thick to be intact, making this condition recognisable [39]. Discolouration at the base of the tail was excluded from the evaluation, as it is often associated with scalding and easily distinguished from actual lesions [38]. In cases where multiple lesions were present on a tail, the most severe one was recorded.

**Figure 1:**
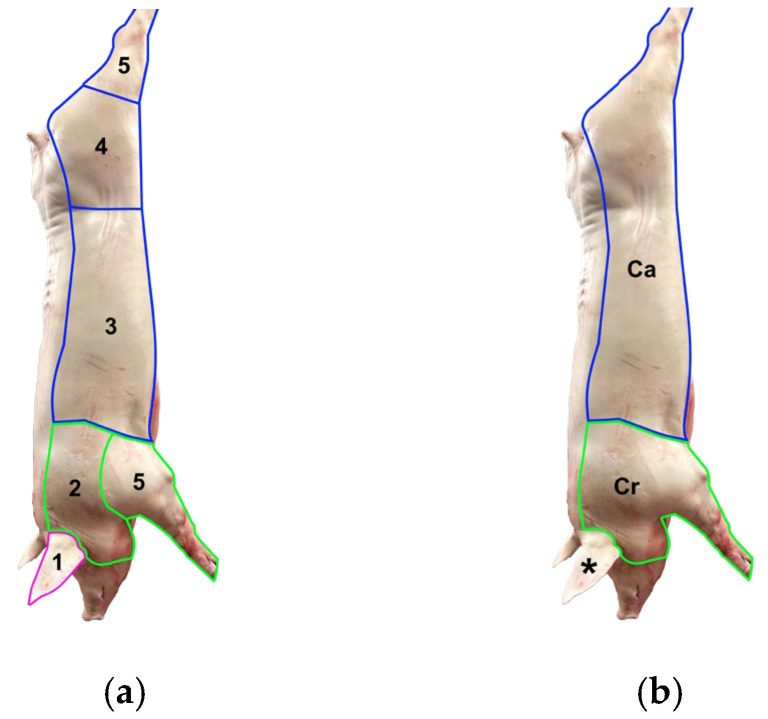
Comparison of skin lesion scoring in carcasses between the Welfare Quality^®^ protocol [3,42] (**a**) and the method used in this study (**b**). (**a**) The carcass is divided into five body areas: ear (1), front (2), middle (3), hindquarters (4) and legs (5). (**b**) Four of these areas are combined into two regions: cranial (Cr) and caudal (Ca), while the ears (*) are assessed separately. The background of the figures has been removed to provide a clearer view of the carcasses.

Traumatic skin lesions were defined as any loss of epidermal integrity, including abrasions, erosions, lacerations, and ulcers extending to deep wounds, as well as bruises, with variable shape (linear, comma-shaped, polygonal) and colour (bright red to yellowish), compatible with traumatic injuries. The size and severity of traumatic skin lesions were graded on a scale of 0 to 3 [22]. The scoring was performed on one side of the carcass, divided into two regions and given two different scores [41]. With the exception of the ears, which were evaluated separately, four of the five body areas (Figure 1a) described in the Welfare Quality^®^ protocol [3,42] for skin lesion assessment were grouped into two regions: cranial and caudal (Figure 1b). The cranial region extends from the cheek to the shoulder and forelimbs, while the caudal region includes the flank and hindquarters.

A score of 1 or 0 was given for the presence or absence of ear lesions, respectively. An ear lesion was defined as erosions, wounds, scars, bruises, missing parts of the ear (excluding man-made ear notches) or ear necrosis (dry, crusty and curled areas) [12].

All the individual AW assessments were aggregated at the batch level in terms of proportional lesion scores, obtained by summing all the individual scores within a batch, then dividing this sum by the number of examined carcasses multiplied by the maximum theoretical score, as follows:

(1) $$\mathrm{Proportional}\;\mathrm{Score}\;=\;\left(\sum_{i}^{n}{\mathrm{score}}_{i}\right)/\left(n\;\times \;\mathrm{Maximum}\;\mathrm{Score}\right)$$

Batch-level proportional scores can range from 0 to 1 and were used in all statistical analyses.

### 2.2. On-Farm Evaluations

Once the farms of origin for all the batches assessed at the abattoir were identified, the presence of AW questionnaires uploaded within the ClassyFarm system was verified. Only those farms having at least one complete questionnaire within the year prior to abattoir evaluations were considered for further analysis.

Two different AW questionnaires are available in ClassyFarm for finishing pig farms: an extended questionnaire covering several AW topics related to fattening stages and a shorter one specifically designed to assess the risk of tail-biting, implemented in accordance with European Commission Recommendation (EU) 2016/336 on the application of Council Directive 2008/120/EC [41,43]. On-farm assessments in ClassyFarm can only be submitted by veterinarians who have completed specific training. Item scores are assigned according to guidelines described in two dedicated manuals [41,43]. The two questionnaires contain 46 and 23 items, respectively, with 16 items in common (Table 2). During the study period, at least one questionnaire was available for 86 out of 108 farms (79.6%). Eighty-four farms (97.7%) were located in northern Italy (Lombardy, Piedmont, Emilia-Romagna and Veneto regions) and two (2.3%) were located in central Italy (Umbria region). Of these farms, 23 uploaded the extended questionnaire, 43 uploaded the tail biting survey and 20 uploaded both.

The items evaluated in each survey are organised in two thematic areas (animal-based measures and resource-based measures), as suggested by the scientific literature [3,4]. The latter area is further divided into: ‘farm management and personnel’, ‘structures and equipment’ and ‘major risks and alarm systems’ [41,43]. To ensure comparability with the AW assessments conducted at the abattoir, only the 16 items common to both questionnaires were included in the analysis (Table 2).

These 16 items are classified as ‘unacceptable’, for conditions that prevent animals from meeting their biological needs; ‘acceptable’, for conditions that generally meet the needs of the animals; and ‘optimal’, for conditions that are significantly better than the minimum legal standards [41,43]. Due to the small proportion of items classified as ‘unacceptable’ (1.38%, 19 out of 1376), these were grouped together with the ‘acceptable’ items into a broader ‘suboptimal’ category.

### 2.3. Statistical Analysis

All data collected for this study, including those extracted from the ClassyFarm database, were managed using Microsoft Excel (Microsoft Corp., Redmond, WA, USA). The relationships between animal welfare at the abattoir and on the farm were investigated, with the batch serving as the experimental unit. Correlations among abattoir scores were tested by Spearman rank correlations. A Spearman correlation test was also used to assess whether tail lesion scores at the abattoir were correlated to the specific ABM item ‘tail-biting lesions’ assessed on-farm. Through a first set of four linear mixed models, we then explored the relationship between each of the abattoir lesion scores (‘tail lesions’, ‘ear lesions’, ‘cranial traumatic skin lesions’ and ‘caudal traumatic skin lesions’) and the proportion of questionnaire items scored as optimal in the following three thematic areas: ‘farm management and personnel’, ‘structures and equipment’ and ‘animal-based measures’. Whenever a thematic area was found to be significantly related to one of the lesion scores, a second linear mixed model was run including all the items within that area as explanatory variables. In all models, farm size and the proportion of animals in the batch with intact tails were included as covariates, while farm ID was included as a random intercept to account for repeated measures from the same farm. All the statistical analyses were performed using R Statistical Software (v4.3.1; R Core Team 2023).

## 3. Results

A total of 18,333 carcasses from 185 batches across 86 farms were assessed at the abattoir, with a median of 105 carcasses per batch (interquartile range, IQR = 16). A median of two (IQR = 1) batches and 160 (IQR = 124) carcasses were evaluated per each farm. Intact tails were present in 3131 of the 18,333 carcasses (17.08%), with a median of 4.4% intact tails per batch (IQR = 15.6%). The 86 farms housed a median of 2450 finishers (IQR = 2510).

### 3.1. Abattoir Welfare Assessment

At the individual level, tail lesions were observed in 3.44% of pig carcasses with non-intact tails and in 38.84% of those with intact tails. Cranial traumatic skin lesions were present in 34.24% and 32.71%, caudal traumatic skin lesions in 34.61% and 32.83%, and ear lesions in 8.06% and 4.25%, respectively.

**Figure 2:**
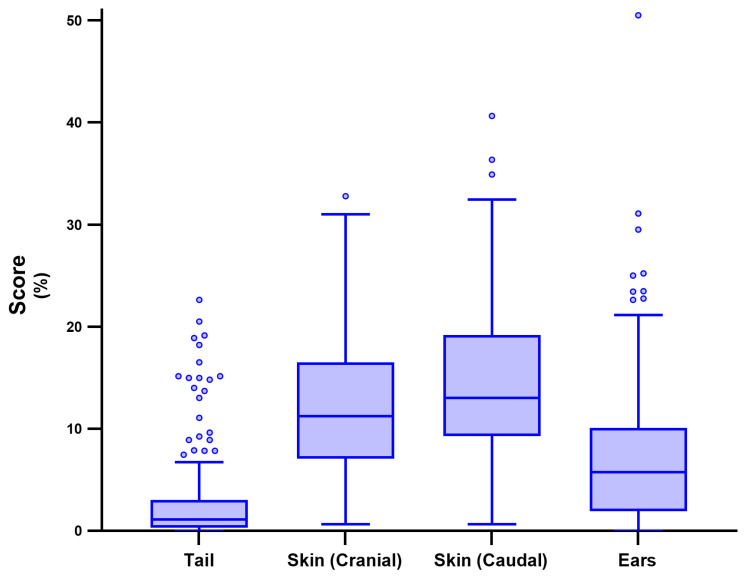
A box and whisker plot (Tukey) showing the distribution of the animal welfare scores (tail, skin and ear lesions) assessed at the abattoir on 18,333 carcasses in 185 batches from 86 Italian heavy pig farms.

Median batch-level proportional scores were: 1.13% (IQR 2.74%) for tail lesions, 11.22% (IQR 2.74%) for cranial traumatic skin lesions, 13.04% (IQR 9.91%) for caudal traumatic skin lesions and 5.77% (IQR 8.18%) for ear lesions. The distribution of these scores is shown in Figure 2.

The proportion of intact tails in a batch was positively correlated with the tail lesion score (ρ = 0.63; *p* < 0.0001) and negatively correlated with the ear lesion score (ρ = −0.32; *p* < 0.0001). Cranial and caudal skin lesion scores were positively correlated with each other (ρ = 0.66; *p* < 0.0001) and with ear scores (ρ = 0.47 and ρ = 0.38, respectively; both *p* < 0.0001).

### 3.2. On-Farm Welfare Assessment

**Figure 3:**
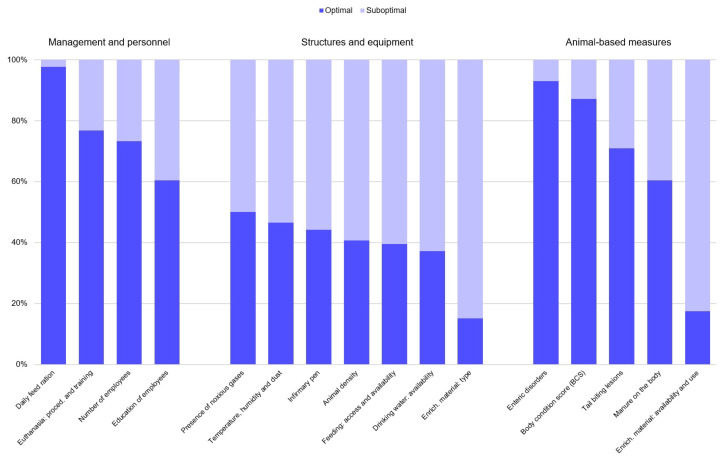
Proportion of optimal (blue) and suboptimal (light blue) evaluations of 16 animal welfare items assessed on 86 Italian heavy pig farms.

On the 86 farms, the median proportion of all optimal items was 56.3% (IQR = 31.3%). The median proportion of optimal items per area was: 75.0% (IQR = 50.0%) for ‘farm management and staff’, 35.8% (IQR = 42.8%) for ‘structures and equipment’ and 60.0% (IQR = 20.0%) for ABMs. The proportions of optimal and suboptimal evaluations for each item are shown in Figure 3.

### 3.3. Integrated Analysis of Farm–Abattoir Relationships

**Figure 4:**
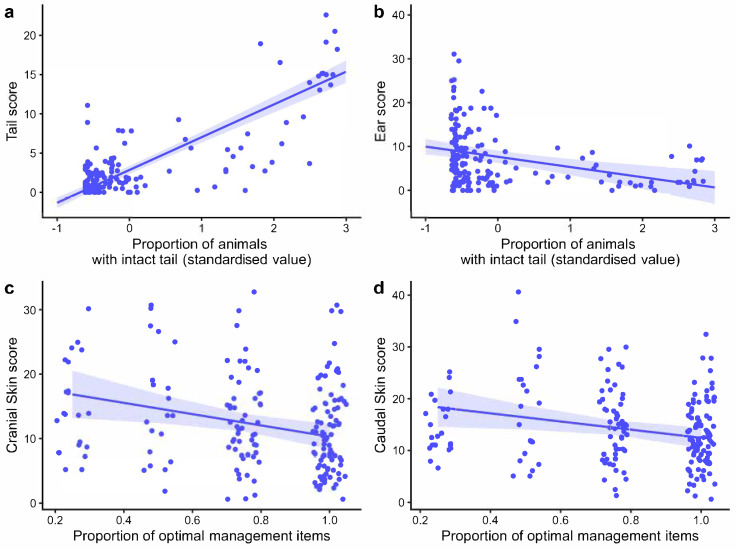
Observed (points) and predicted (lines) relationship of (**a**) tail scores and proportion of animals with intact tails; (**b**) ear scores and proportion of animals with intact tails/proportion of optimal management items; (**c**) cranial skin scores and proportion of optimal management items; (**d**) caudal skin scores and proportion of optimal management items. Bands represent 95% CIs of the predictions.

Tail scores at the abattoir strongly increased with the proportion of intact tails within the batch (parameter estimate ± SE: 4.16 ± 0.22, *p* < 0.0001; Figure 4a) but showed no relationship with any of the thematic areas from on-farm assessments (all *p* > 0.05) nor any significant correlation with on-farm ‘tail -biting lesions’ assessment. Cranial and caudal traumatic skin lesions were significantly related to the on-farm management assessment, with higher scores associated with suboptimal evaluations (−9.60 ± 3.24, *p* = 0.004; and −9.32 ± 3.39, *p* = 0.008, respectively; Figure 4c and Figure 4d). In particular, cranial skin scores were negatively related with staff training (−4.77 ± 1.56, *p* = 0.003), while caudal lesions were negatively related to both staff training (−3.98 ± 1.54, *p* = 0.012) and feed management (−16.26 ± 5.26, *p* = 0.002). Likewise, ear scores were negatively associated with on-farm management (−7.95 ± 3.27, *p* = 0.018), but they also showed a negative relationship with intact tails (−2.32 ± 0.58, *p* = 0.0001; Figure 4b), with lower proportions of animals with intact tails in a batch resulting in higher ear scores.

## 4. Discussion

This study was carried out on intensive pig farms in Italy that use a distinctive fattening system involving slaughtering pigs at over nine months of age and with a live weight of 160–170 kg. These pigs are considerably older and heavier than those usually reared for slaughter around the world [44]. Pig farming is primarily concentrated in northern Italy and is mostly dedicated to producing Protected Designation of Origin (PDO) and Protected Geographical Indication (PGI) products. Such production requires specific nutritional strategies and a prolonged rearing phase, which may influence welfare risks, as well as the type and chronicity of lesions observed at slaughter [45]. In particular, the present study examines the relationship between iceberg indicators of AW (tail, skin and ear lesions) at the abattoir and AW assessments carried out on the farms of origin, using data from the Italian national surveillance system (ClassyFarm). There is still limited information connecting post-mortem lesions with on-farm rearing conditions in heavy pigs, especially studies combining detailed on-farm welfare protocols with carcass-level scoring.

Tail lesions are considered a valid indicator of pig welfare [16], providing valuable insights for the assessment of AW during abattoir evaluations [5]. In this study, the low median tail lesion score of just over 1% was due to the generally low incidence of tail lesions, particularly severe ones. However, substantial variation was observed between the batches (Figure 2), as well as significant differences depending on whether tails were predominantly intact or not. These large variations confirm the multifactorial nature of tail-biting behaviours, which may be both challenging to predict and to control [46,47]. At the individual level, tail lesions were observed in 38.8% of pigs with intact tails and in 3.4% of those with non-intact tails. Similar differences have been reported in other European countries. For example, the prevalence in docked pigs ranges from 1.6 to 3.1% in Ireland [8,38,48] to 25.4% in Germany [40], whereas in undocked pigs it ranges from 7.1% in Sweden [49] to 36.8% in Switzerland [50]. However, these studies mainly involved pigs slaughtered at 80–110 kg. Data on heavy pigs (160–170 kg) are more scarce due to their limited production outside Italy [51]. An earlier Italian study on heavy pigs reported a similar prevalence of tail lesions in undocked pigs, but only 0.2% in docked pigs [32]. This variability within and between countries may be partly explained by differences in production and environmental conditions. Methodological factors may also contribute, including inconsistent scoring of lesion severity, different definitions of intact/undocked tails and subjective variability in evaluations [8,49]. In addition, the stage of carcass processing at which scoring is performed (i.e., before or after scalding) may influence the results [38]. These factors complicate comparisons between studies and emphasise the need for standardised tail-lesion scoring methods. Moreover, despite the long-standing recognition of the need for standardised protocols to enable cross-study comparability and actionable farmer feedback, their implementation has been limited in pig production [15]. Unlike in broiler production, for example, where carcass lesions can result in penalties or condemnation [52], pigs usually lack comparable economic incentives, which may have hindered progress towards such harmonisation.

A positive relationship was observed between the proportion of carcasses with intact tails in a batch and the frequency and severity of tail lesions. This likely results from rearing pigs with undocked tails in environments that fail to meet their behavioural needs. These findings are consistent with the existing literature showing that undocked pigs exhibit a higher prevalence and severity of tail lesions, both at the abattoir and on-farm [12,17,20,32,51,53]. Reducing the incidence of tail biting [18] and tail docking does not necessarily improve overall AW [54,55] and often redirects aberrant behaviours to other areas of the body [56] such as ears [17,30]. Preventive strategies should instead prioritise enhancing the pigs’ environment to meet their behavioural needs, thereby reducing the need for tail docking [57].

No relationship was observed between abattoir tail scores and the corresponding on-farm ABM, in contrast with a previous study conducted in Ireland [21]. This discrepancy may be partly explained by methodological differences between abattoir and on-farm evaluations. On-farm tail lesions were assessed qualitatively at the herd level (optimal/suboptimal) by the herd veterinarian based on severity thresholds that varied depending on whether the pigs had been docked [41,43]. Conversely, individual-level quantitative data were collected at the abattoir by two authors (L.S., M.R.), using a uniform scoring system for both intact and non-intact tails. Furthermore, assessing tail lesions in live animals is inherently more challenging than in carcasses due to reduced visibility caused by dirt, poor lighting and animal movement within pens [6]. Temporal mismatches between evaluations could also affect relationships between farm and abattoir data, as previously described by a Chilean study which found only weak links without a longitudinal tracking of the assessed pigs [58].

The lack of any relationship between tail lesion scores and other on-farm resource- and animal-based evaluations may be explained by the complexity and multifactorial nature underlying tail-biting outbreaks, which makes a comprehensive identification of on-farm risk factors challenging [16,20]. Even in farms with optimal conditions, tail-biting outbreaks can still occur, reflecting the unpredictable nature of this behaviour [19,20] and making it difficult to completely abandon tail-docking practices. Comparing these findings with previous studies is also difficult due to differences in methodology. For instance, some studies have compared different ABMs based on other welfare assessment protocols (e.g., Welfare Quality^®^) [3], while others have focused on the loading, transport and lairage phases [25,26,34,35,36] or used physiological measures as stress indicators [59,60].

Although not as prominent as tail lesions [11], traumatic skin lesions are another iceberg indicator of pig welfare [16]. However, similar to tail lesions, no internationally shared scoring system is available. In the present study, cranial and caudal traumatic skin lesions were not assessed by dividing the carcass into equal halves, nor in the four regions used in other protocols [12], but rather by considering a compromise between the ethogram of the pig, the way traumatic skin lesions can occur and the feasibility in a high-speed slaughter line. Skin lesion scores were positively correlated with each other and with ear lesion scores, suggesting a possible common cause. Previous studies have reported that traumatic skin lesions resulting from pre-slaughter aggression primarily affect the head and shoulder areas, while posterior lesions mainly occur due to contact with inadequate structures, rough handling or by pigs mounting and scratching each other’s backs with front claws [22,25,61]. This behaviour is common in overcrowded pens, during loading/unloading or along stunning chutes [61]. In addition to the location, severity and extent of traumatic skin lesions, some studies also consider wound shape and colour [22,61]. The absence of detailed transport data also complicates the attribution of lesion origins to either on-farm conditions or pre-slaughter handling. Traumatic skin lesions, particularly fresh scratches on cranial regions, can develop during loading, transport and lairage due to fighting, mounting behaviour or contact with vehicle structures [15]. Nevertheless, the positive relationship between cranial and caudal traumatic skin lesions and, in particular, their association with suboptimal farm management, suggest the presence of a farm-level component. Furthermore, transport stress may exacerbate aggressive behaviour in pigs from poorly managed farms. To better understand the relationship between traumatic skin lesions observed in different areas of the carcass, future studies could incorporate assessments of lesion chronicity. For instance, the presence of fresh cranial lesions and older caudal lesions on the same carcass could suggest different underlying causes. Additionally, combining farm-level data with national transport databases and meat inspection records, as proposed in data-driven welfare surveillance models [62,63], could enhance the granularity and temporal resolution of welfare assessments.

Optimal management practices, particularly staff training and appropriate feed rations (for caudal lesions), significantly reduced the prevalence of traumatic skin lesions observed at the abattoir. These findings are consistent with studies indicating that suboptimal management practices and inadequate staff training can lead to increased aggression among pigs, resulting in more severe skin injuries [17,64].

Ear lesions, like tail and traumatic skin lesions, represent another important AW indicator that can be assessed at the abattoir [17]. The prevalence of these lesions was twice as high in pigs with non-intact tails compared to those with intact tails (8.6% vs. 4.6%), similar to the results reported in previous Italian studies [12,17]. There was a significant positive relationship between ear lesion scores and the proportion of pigs with non-intact tails, which is consistent with previous studies documenting a substitution effect between tail and ear biting [17,28]. In herds where tail docking is practised without concurrent improvements in housing conditions, pigs may express distress through behaviours other than tail biting, such as inflicting ear lesions through biting or fighting [17,53]. A negative association was also observed between the ear lesions and optimal management practices. This result confirms the value of this ABM as an indicator of AW, especially considering that ear lesions are easily observed at the abattoir and may reflect long-term behavioural problems [65].

In this study, we did not find any relationship between herd size and AW, suggesting that there are other, more important factors influencing AW. This finding is consistent with previous studies that have found little to no evidence of an effect of farm size on AW [66,67]. However, it is important to note that our study only included intensive farms, typically housing over 1000 finishers, with a fairly standardised type of production, all delivering to the same industrial abattoir. It cannot be excluded that a larger, more heterogeneous sample would also show an effect of farm size on AW.

While the assessment of AW indicators at the abattoir offers several advantages, it also has some limitations that may affect its accuracy, such as the exclusion of animals that died or were euthanised on the farm [68,69]. In addition, the high speed of carcass processing in industrial abattoirs can make it difficult to distinguish healed tail lesions from docked tails [69,70], potentially leading to an underestimation of AW problems. To improve accuracy, abattoir observations should still be complemented by on-farm assessments [68]. Comparability is another important limitation affecting pig welfare studies in general, as reliable comparisons across countries require robust, harmonised assessment methods [15]. Ideally, these would be based on international standards, which could improve consistency [4]. Due to limited resources, it was not feasible to follow the same pigs from farm to slaughter, which may have affected the analysis of the impact of farm conditions on slaughter outcomes. Furthermore, it was not possible to evaluate other potential influencing factors in the present study, such as specific genetic lines, sex and potential seasonal variations. These factors should be considered in future research. On-farm assessments are also carried out by different herd veterinarians, potentially introducing bias. The small sample size, particularly of farms rearing pigs with undocked tails, may have further limited the generalisability of this study. Finally, implementing systematic assessments of ABMs in industrial abattoirs may be impractical due to training costs, high slaughter line speeds and limited staff. Artificial intelligence may offer a solution in the future, potentially increasing data objectivity and reliability [65].

## 5. Conclusions

This study examines relationships between three abattoir-based welfare indicators (tail, skin and ear lesions) and on-farm assessments in Italian heavy pig production. While tail lesions were more common in pigs with intact tails, confirming the challenges of rearing undocked pigs, a substitution effect between tail and ear lesions in docked pigs was also observed. This confirms that tail docking without concurrent environmental improvements, such as providing optimal enrichment materials, can redirect negative behaviours rather than address their root causes.

Cranial and caudal skin scores were related to each other and to ear scores, suggesting a multifactorial origin associated with pre-slaughter handling and husbandry practices. Indeed, optimal management practices resulted in lower, better scores. However, the extended rearing period in heavy pig production may amplify chronic welfare issues, as well as complicate the temporal attribution of lesions.

The lack of relationships among some of the assessed indicators, notably for tail biting, could be the result of the complex underlying factors influencing AW and differences in evaluation methods. Furthermore, they could be influenced by evaluator bias and temporal mismatches. Nevertheless, these issues are unavoidable in real-world surveillance settings, which emphasises the importance of integrated surveillance in providing useful feedback to farmers and veterinarians.

Future research should prioritise tracking the same pigs from farm to slaughter, assessing lesion chronicity and collecting and integrating transport data systematically, as well as incorporating precision livestock farming technologies, in order to further expand knowledge on this topic.

Finally, standardised, harmonised protocols are essential for cross-study comparability and for providing farmers with actionable feedback to improve animal welfare in intensive pig production.

## Figures and Tables

**Table 1:** Definition of the scoring system and description of the lesions evaluated at abattoir.

Lesion	Score	Description
Tail lesions	0	No visible lesion
	1	Skin damage with reddish discoloration (scratches, bites and hematomas), with no tissue missing
	2	Minor damage with loss of tissue < 2 cm, not fully healed
	3	Major damage with loss of tissue > 2 cm, not fully healed
Traumatic skin lesions	0	None or a short (2–3 cm) superficial lesion
	1	Some superficial lesions, clearly marked or up to three short (2–3 cm) and deep lesions
	2	Clear deep and/or long lesions (>3 cm) including much superficial lesions or circular areas
	3	Much deep lesions
Ear lesions	0	Absence of lesions
	1	Presence of lesions (scratches, bites, bruises)

**Table 2:** List of the 16 on-farm evaluation parameters common to both the ClassyFarm animal welfare questionnaires available for finisher pigs (extend welfare evaluation and tail-biting risks assessment).

Measure Type	Area	Item
Resource-based	Farm management and personnel	Number of employees
		Education of employees
		Euthanasia: procedures and training
		Daily feed ration
	Structures and equipment	Feeding: access and availability
		Drinking water: availability
		Infirmary pen
		Animal density
		Temperature, humidity and dust
		Presence of noxious gases
		Enrichment material: type
Animal-based	Animal-based measures	Enrichment material: availability and use
		Manure on the body
		Tail-biting lesions
		Body condition score (BCS)
		Enteric disorders

## Data Availability

The data presented in this study are available on request from the corresponding author due to their sensitive nature and only after anonymisation of the abattoir and farms included in this study.

## Supplementary Materials

The following supporting information can be downloaded at: https://www.mdpi.com/article/10.3390/vetsci13040361/s1, Table S1: Evaluation of abattoir-based measures (tail, skin and ear lesions) in pigs: description of the scoring systems and examples in heavy pigs.
