# Supplementary material for: Comparative Analysis of Abattoir-Based Measures and On-Farm Pig Welfare Indicators in Italian Fattening Heavy Pigs
Source: Vet Sci. 2026 Apr 8;13(4):361. doi: 10.3390/vetsci13040361 (PMC13120301; doi:10.3390/vetsci13040361)
Supplement: Supplementary file 1 [file vetsci-13-00361-s001.zip › vetsci-4234803-supplementary.pdf]

Comparative analysis of abattoir-based measures and on farm pig welfare indicators in Italian fattening heavy pigs

Lucia Scuri, Matteo Recchia, Federico Scali, Claudia Romeo, Antonio Marco Maisano, Giovanni Santucci, Adriana Ianieri, Sergio Ghidini and Giovanni Loris Alborali

Table S1: Evaluation of abattoir-based measures (tail, skin and ear lesions) in pigs: description of the scoring systems and examples in heavy pigs.

Lesion	Score	Description	Example
**Tail lesions**	0	No visible lesions	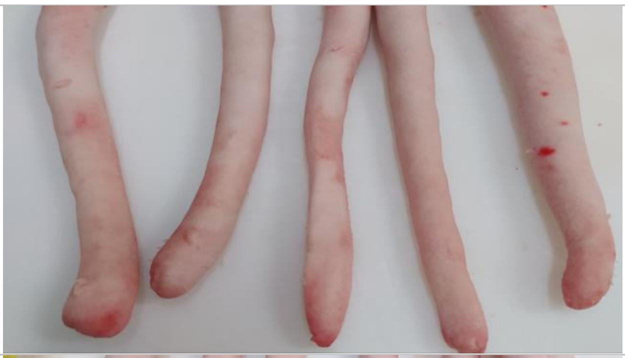
	1	Skin damage with reddish discoloration (scratches, bites and haematomas), with no tissue missing	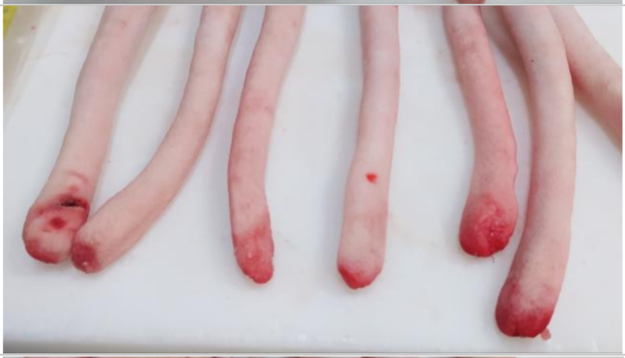
	2	Minor damage with loss of tissue < 2 cm, not fully healed	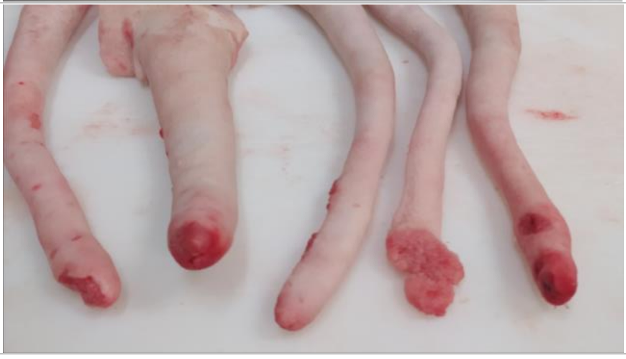

	3	Major damage with loss of tissue > 2 cm, not fully healed	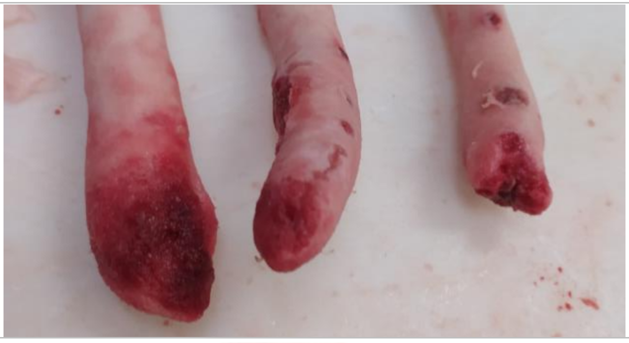
**Skin lesions**	0	None or a little superficial damage	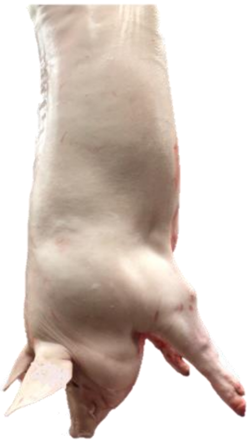
	1	Some superficial damage, clearly marked or up to three short (2-3 cm) and deep	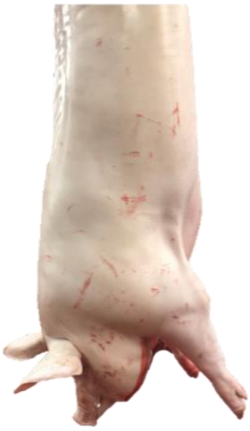
	2	Clear deep and/or long damage (> 3 cm), including much superficial damage or circular areas	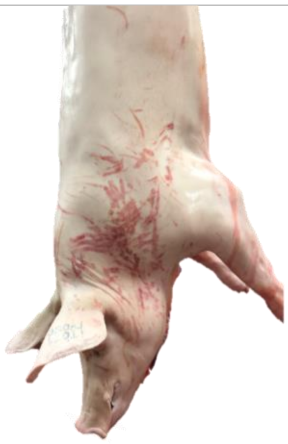

	3	Much deep damage	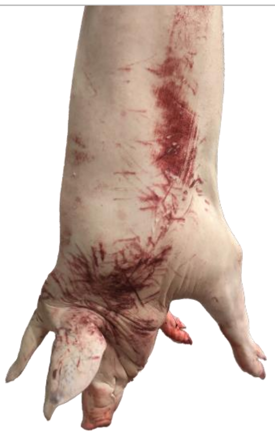
**Ear lesions**	0	Absence of lesions	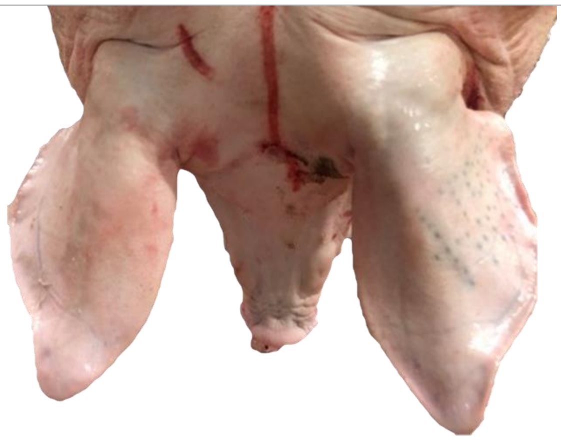
	1	Presence of lesions (scratches, bites, bruises)	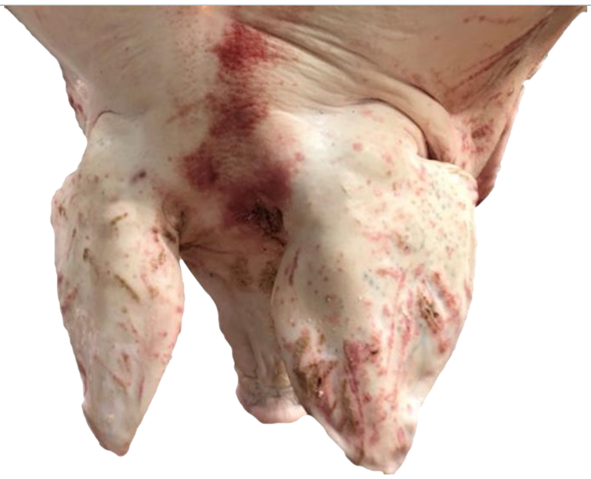
